# Cardiac Tumors

**DOI:** 10.5402/2011/208929

**Published:** 2011-05-26

**Authors:** Ioannis A. Paraskevaidis, Christos A. Michalakeas, Constantinos H. Papadopoulos, Maria Anastasiou-Nana

**Affiliations:** ^1^Second Department of Cardiology, Athens University Medical School, Attikon University Hospital, 1 Rimini St, 12462 Athens, Greece; ^2^Second Department of Cardiology, Hellenic Red Cross Hospital, 1 Erythrou stavrou St, 11526 Athens, Greece

## Abstract

Cardiac tumors represent a relatively rare, yet challenging diagnosis. Secondary tumors are far more frequent than primary tumors of the heart. The majority of primary cardiac tumors is benign in origin, with primary malignant tumors accounting for 25% of cases. Metastatic tumors usually arise from lung, breast, renal cancer, melanomas, and lymphomas. Clinical manifestations of cardiac tumors depend on the size and location of the mass and the infiltration of adjacent tissues rather than the type of the tumor itself. Echocardiography is the main diagnostic tool for the detection of a cardiac mass. Other imaging modalities (C-MRI, C-CT, 3D Echo) may offer further diagnostic information and the establishment of the diagnosis is made with histological examination. Management depends on the type of the tumor and the symptomatology of the patient.

## 1. Introduction

Cardiac tumors are a challenging and bizarre clinical situation. They are differentiated into primary and secondary (metastatic). The prevalence of primary cardiac tumors is 0.001–0.03% in autopsy series [1]. Seventy-five per cent of primary tumors are benign in origin, with myxoma being the most frequent over 50% of cases. From the remaining 25% of malignant cardiac tumors, most frequent are cardiac sarcomas. Secondary tumors are from 20- to 40-fold more common than primary tumors, and 15% of patients suffering from any form of cancer exhibit metastases in the heart.

## 2. Classification

### 2.1. Primary Cardiac Tumors

#### 2.1.1. Benign Tumors

**Myxoma**

Myxoma represents the most frequent benign tumor in adult population [2]. It accounts for 25% of all cardiac tumors, more than 50% of benign cardiac tumors, and it affects mainly women and age groups of 30 to 60 years old. Myxomas are thought to originate in undifferentiated and totipotent mesenchymal stem cells [3]. Seventy-five per cent of myxomas are located in the left atrium (arising from the fossa ovalis of the interatrial septum), 20% in the right atrium and the remaining 5% in the ventricles. Myxomas usually have a narrow base of attachment (pedicle) to the cardiac wall and their composition is heterogeneous, consisting of areas with hemorrhage, necrosis, cyst formation, fibrosis, and calcification. In approximately 7% of cases, myxomas are familial, with most pronounced case being the Carney complex. This syndrome is an autosomal dominant inherited disease, where multiple myxomas, extracardial myxomas (breast, skin), schwannomas, abnormal skin pigmentation and endocrine overactivity or endocrine tumors, may coexist [4]. In cases of atrial myxomas in the setting of the Carney complex, relapses are frequent, and close monitoring of the patients is essential.

**Lipomas and Lipomatous Hypertrophy of the Interatrial Septum**

Lipomas represent the second most frequent primary tumor, and they are usually located in the subepicardium, in the left ventricle, the right atrium, and the interatrial septum. Often they are asymptomatic; however, they may cause arrhythmias, conduction system disturbances, and symptoms of heart failure, especially in cases where they reach a large size [5]. Lipomatous hypertrophy of the interatrial septum is the result of hyperplasia and the accumulation of adipose tissue on the interatrial septum (except for the fossa ovalis area), and it affects mainly the elderly and obese male patients [6].

**Papillary Fibroelastoma**

Papillary fibroelastoma is the most common tumor of the cardiac valves, accounting for 75% of valvular tumors. Papillary fibroelastoma usually affects the elderly (age range 60 ± 16) [7]. It is generally small in size (<1 cm), and it affects mainly the aortic or the mitral valve, even though tricuspid and pulmonary valves may also be affected [8].

**Rhabdomyoma**

Rhabdomyoma represents the most common cardiac tumor in children. Rhabdomyomas are usually multiple, and they affect the right and the left ventricle likewise. They may cause mechanical complications, such as obstruction of the right ventricular outflow tract. It is also common for rhabdomyomas to regress spontaneously after birth [9].

**Fibroma**

Fibroma is the second most common cardiac tumor of childhood, although it may also affect the adult population. Fibromas are intramural tumors, located in the left ventricle, mainly in the intraventricular septum, and they are often mistaken for hypertrophic cardiomyopathy or apical thrombus.

**Other Primary Benign Tumors**

Angiomas, teratomas, and mesotheliomas are rare tumors (accounting for 7% of cardiac tumors), and they affect mainly children. Teratomas are located in the pericardium, and they may lead to constrictive pericarditis.

#### 2.1.2. Malignant Tumors

 Primary malignant tumors are relatively rare (accounting for 25% of primary cardiac tumors); they affect ages from 30 to 50 years old, and they are usually sarcomas (angiosarcoma, rhabdomyosarcoma, leiomyosarcoma, liposarcoma, osteosarcoma, fibrosarcoma, and malignant fibrous histiocytoma). Most angiosarcomas are usually located in the right chambers of the heart [10], whereas other sarcomas affect the left atrium more frequently [11]. Malignant tumors confer a poor prognosis by extensively infiltrating the myocardium, causing obstruction of intracardial flow and producing metastases.

### 2.2. Secondary (Metastatic) Cardiac Tumors

 Metastatic cardiac tumours are far more frequent (approximately from 30- to 40-fold) than primary tumors of the heart [12]. They usually arise from melanomas, lung, breast, and renal cancer, as well as lymphomas. Metastases may originate from blood dissemination of cancer cells, direct extension via adjacent tissues, or propagation via the superior or the inferior vena cava to the right atrium. Pericardium is most often affected, resulting in pericardial effusion which may contain masses comprising either cancer cells or blood clots and fibrin.

 The “carcinoid syndrome” leads to lesions usually in the right heart chambers. It affects mainly the cardiac valves with deposition of fibrous plaques, leading to thickening, shortening, and motion restraint of the tricuspid and/or the pulmonic valve and subsequent regurgitation or stenosis of these valves.

## 3. Diagnostic Evaluation (Figure 1)

 Diagnosis and differential diagnosis of cardiac tumors often presents a challenge for the physician. Cardiac tumors, either benign or malignant, are difficult to be diagnosed due to their rarity, variety, and nonspecificity of the symptoms that they may cause. Patient's history, clinical examination, and blood tests rarely lead to an immediate diagnosis of the tumor; therefore, suspicion of this condition is critical for the correct and timely diagnosis of a cardiac tumor. Furthermore, beyond the performance of imaging techniques (discussed below), histological evaluation via biopsy is essential for the final diagnosis to be established.

### 3.1. Clinical Manifestations

 Clinical manifestations of cardiac tumors are differentiated in to four categories: systematic manifestations, embolic events, cardiac manifestations, and finally manifestations due to metastases.

 The *systematic symptoms* and laboratory findings may resemble those of vasculitis and connective tissue diseases and may disorientate from the correct diagnosis. Fever may be present, as well as fatigue, arthralgia, rush, and the Raynaud phenomenon. The laboratory tests may reveal anemia, elevated white blood cell count and platelet count, thrombocytopenia, hypergammaglobulinemia, and elevated erythrocyte sedimentation rate (ESR). Systematic manifestations are attributed to secretion of various factors from the tumor cells (Interleukin 6, Endothelin). 

 Cardiac tumors may be the cause of *pulmonary embolism or peripheral embolism* due to the embolism of tumor cells or thrombi formed on the tumor surface. Predisposition to embolic episodes depends mainly on the type of the tumor, its location (intramural or intracardial), and the fragility of its surface. Small tumors with a friable appearance have a higher chance of embolization.

 Regarding *cardiac manifestations*, these depend mainly on the anatomic location, the size of the tumor, and the infiltration of adjacent tissues, rather than the histological type of the tumor. Cardiac manifestations may be caused by direct obstruction of cardiac or valve function, interruption of coronary flow, interpolation on the electrophysiology of myocardial contraction, and induction of pericardial effusion. Intramural tumors rarely produce symptoms, especially if their size is small. They may cause arrhythmias or disturbance of the conduction system, and in cases of large tumors, they may cause obstruction of the right or left outflow tract or compression of the cardiac chambers. Intracardiac tumors may exert severe clinical manifestations. Characteristically, myxomas of the left atrium may cause symptoms of mitral obstruction through prolapse via the mitral valve (syncope depending on change of patient's position, dyspnoea, etc.). Tumors located in the right chambers of the heart may cause signs and symptoms of right-sided heart failure.

 Regarding cardiac manifestations due to *metastatic disease* from other organs, these usually affect the pericardium (55%, pericardial effusion, cardiac tamponade, or constrictive pericarditis) and less often the myocardium, the endocardium (heart failure, arrhythmias), and the venae cavae (obstruction). In general, the presentation of cardiac manifestation in a patient suffering from cancer is usually attributed either to cardiotoxicity because of chemotherapy [13] or occurrence of cardiac metastases.

 Finally, primary cardiac tumors can cause symptoms through metastases to other organs. Most frequently, sarcomas metastases affect lungs, brain, and bones.

### 3.2. Imaging Techniques

 The imaging techniques that are used when there is a suspicion for the occurrence of a cardiac tumor, as well as for the differential diagnosis of other cardiac masses like vegetations and thrombi, are mainly echocardiography, magnetic resonance imaging (MRI), and computed tomography (CT) of the heart. Chest X-ray can offer indirect findings from the enlargement of cardiac chambers, the occurrence of calcification, or pericardial effusion. 

#### 3.2.1. Echocardiography

 Echocardiography represents a substantial imaging technique for the detection of cardiac tumors with a high sensitivity and specificity (90% and 95%, resp.), and it can be easily performed at the patient's bedside [14]. *Transthoracic echocardiography (TTE)* can depict the shape, the size, the extent, and the mobility of the tumor, as well as, its location and its relation to adjacent cardiac structures, the width of adherence to the cardiac wall, and the hemodynamic consequences that it may confer to cardiac function [15]. *Transesophageal echocardiography (TEE)* is superior to TTE, since one can avoid transducer contact with the thoracic wall and the lungs and achieve a better visualization and identification of small tumors (<5 mm) and tumors localized at the posterior cardiac segments [16]. It should be performed when there is a low quality of TTE image due to poor acoustic window or when serious clinical questions regarding the nature of a mass remain unanswered. However, the echo field remains narrow and no information regarding the composition and the perfusion of a mass is given.

 Of interest are newer echocardiographic techniques that may aid in the differential diagnosis of cardiac tumors from other cardiac masses. *Contrast echocardiography* helps in the differential diagnosis between tumor and thrombus by examining tissue perfusion [17]. In contrast to thrombi, malignant tumors or tumors rich in vascularity, in general, appear with an intense enhancement of the echocardiographic image when contrast medium is administered, and therefore, contrast echo leads to an accurate diagnosis. Benign cardiac tumors (i.e., myxomas) exhibit sparse vascularity, and medium enhancement of the echocardiographic signal appears sometimes even lower to that of proximal myocardium. Therefore, because of their sparse vascularity, the differential diagnosis between myxomas and thrombi, with the use of contrast echocardiography, is less reliable in comparison to malignant tumors [18].

*Three-dimensional echocardiography (3D Echo)* contributes mainly to an improved assessment of the shape, the size, the mobility of a tumor and its relationship regarding adjacent structures, by making use of the wider imaging range that this technique provides [19]. Improvement of this technique in order to provide better temporal and spatial resolution is expected in the near future.

#### 3.2.2. Magnetic Resonance

 The use of cardiac magnetic resonance (C-MRI) with its wide imaging range allows for a better assessment of the tumor relation to adjacent structures, in order for a surgical resection technique to be designed. It also allows the detection of myocardial infiltration by the tumor or expansion of the mass to the pericardium or to adjacent structures [20]. C-MRI may also contribute to the characterization of the composition of the tumor by studying the signal in T1- and T2-weighted images, as well as the enhancement of the signal after gadolinium administration. Recent technologic advances in cardiac MRI have resulted in the rapid acquisition of images of the heart with high spatial and temporal resolution and excellent myocardial tissue characterization [21]. Administration of contrast medium helps differentiate a cardiac tumor from thrombus formation or blood flow artifacts [22]. 

#### 3.2.3. Computed Tomography

 Cardiac computed tomography (C-CT) can also provide useful information, due to its high resolution and its ability to accurately depict cardiac morphology without limitations because of acoustic windows. Disadvantages of the method include the use of radioactivity and of nephrotoxic contrast mediums. C-CT provides less information regarding characterization of tissues in comparison to C-MRI; however, it can provide some information regarding the nature of the tumor by measuring X-ray attenuation and possible tumor expansion to adjacent tissues. Multidetector computed tomography (MDCT) is useful for the evaluation of calcification and fat content within a mass. Furthermore, the high spatial resolution of MDCT is beneficial to define small lesions, making this technique a useful tool for the staging of malignant tumors [23].

### 3.3. Histological Evaluation

 The diagnosis of cardiac tumors and the estimation of their grade cannot be made with the use of imaging methods only, therefore histological confirmation is necessary. This can be achieved with minimally invasive techniques such as cytological examination of pericardial or pleural fluid or echocardiographically aided percutaneous or transvenous cardiac biopsy. In cases where diagnosis cannot be established, biopsy via thoracoscopy or even thoracotomy may be needed.

## 4. Management

 Therapy of benign primary cardiac tumors is surgical resection, and the urgency to intervene is determined by the symptoms of the patient and the type of the tumor. Regarding myxomas, immediate surgical resection is indicated, regardless of symptoms, because of the high risk of embolic and cardiac complications. Surgical resection of myxomas confers good results (3% possibility of relapse) and is accompanied with small rates of periprocedural mortality (<5%). Papillary fibroelastomas are surgically removed in cases of large (>1 cm) and/or mobile tumors. In cases of small, immobile tumors in the left ventricles, conservative management and close follow-up may be advocated. However, because of their fragile nature, papillary fibroelastomas carry a high risk of embolic complications; therefore, most authors propose surgical management even in asymptomatic patients [24]. Lipomas and lipomatous hypertrophy of the interatrial septum are surgically managed only in cases of serious hemodynamic compromise. Rhabdomyomas usually do not require surgical management, since they tend to regress spontaneously. 

 Regarding malignant primary cardiac tumors, their prognosis is dismal, since they tend to infiltrate the myocardium rapidly, cause obstruction of the cardiac chambers and produce metastases. Management of choice in sarcomas is surgical resection, with poor results and high rates of relapse [25]. Chemotherapy is used as an adjuvant to help decrease tumor size and facilitate surgical resection, or in cases of nonoperable or metastatic sarcomas. In spite of the aforementioned therapeutic attempts the survival rate in cases of sarcomas does not usually exceed one year. In cardiac lymphomas, systematic chemotherapy with or without radiotherapy is usually undertaken.

 Finally, regarding cardiac manifestations due to metastatic extracardiac cancer, priority is given to the management of the primary focus of the disease and the cardiovascular complications that are manifested (i.e., percutaneous balloon pericardiotomy in cases of cardiac tamponade, radiotherapy and chemotherapy in cases of tumors that obstruct flow in the venae cavae, etc.).

## Figures and Tables

**Figure 1:**
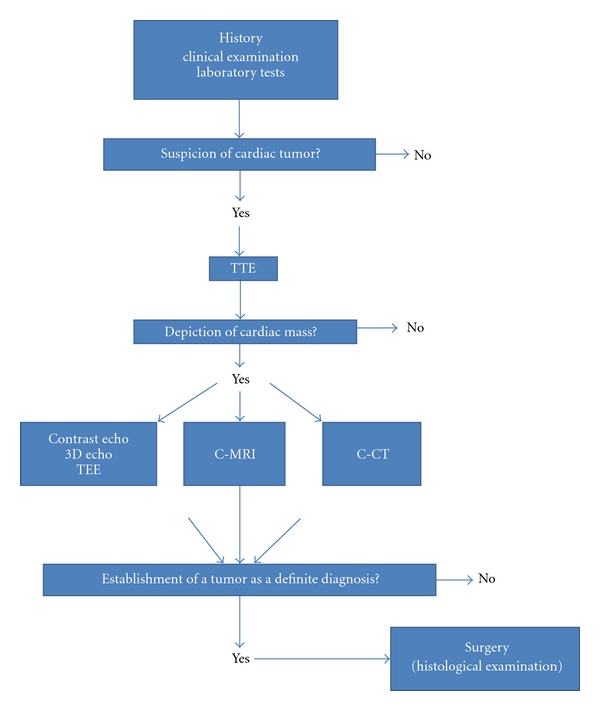
Algorithm for the detection and differential diagnosis of a cardiac tumor. TTE: Transthoracic echocardiography, TEE: Transesophageal echocardiography, 3D Echo: Three dimensional echocardiography, C-MRI: cardiac magnetic resonance, C-CT: Cardiac computed tomography.
